# Treatment of Latent Tuberculosis Infection in a Kawasaki Disease Patient Receiving Anti-Tumor Necrosis Factor Alpha (TNFα) Therapy

**DOI:** 10.7759/cureus.70407

**Published:** 2024-09-28

**Authors:** Koji Yokoyama, Toshinari Yakuo, Mitsukazu Mamada

**Affiliations:** 1 Pediatrics, Japanese Red Cross Wakayama Medical Center, Wakayama, JPN

**Keywords:** immunosuppressive treatment, infliximab, kawasaki disease, latent tuberculosis infection, rifampicin

## Abstract

Kawasaki disease (KD) is a common vasculitis syndrome that mainly affects children. Latent tuberculosis infection (LTBI) is a tuberculosis (TB) infection without signs or symptoms. Here, we report the case of a boy who traveled from abroad, was diagnosed with KD at our hospital, and underwent multidisciplinary treatment, including anti-TNFα therapy. Subsequently, the patient was found to have LTBI and was administered anti-TB treatment. To the best of our knowledge, this is the first reported case of LTBI in a KD patient receiving immunosuppressive therapy containing anti-TNFα.

## Introduction

Kawasaki disease (KD) is one of the most common vasculitis syndromes. It has an unknown etiology and generally occurs in children, with the highest incidence in East Asia, particularly in Japan, South Korea, and Taiwan [1]. The most serious complications of KD are coronary artery lesions, including myocardial infarction and coronary artery aneurysms [2]. Treatment options for KD are increasing [3]. The latest standard initial treatment for KD in Japan is intravenous immunoglobulin therapy (IVIG). Additional treatments include IVIG re-administration and the administration of infliximab (IFX), prednisolone, or cyclosporin A (CsA) [4]. Tuberculosis (TB) is an infectious disease usually caused by *Mycobacterium tuberculosis* (M. tb). The number of people newly diagnosed with TB continues to increase worldwide [5]. In the US, approximately 13 million people have latent TB infection (LTBI), defined as people infected with M. tb who do not have symptoms and do not transmit the disease. Without treatment, approximately 5-10% of LTBI patients will develop active TB disease during their lifetime [6]. However, in children and adolescents, the risk of developing TB disease after infection is much greater, estimated at 15% within five years and 33% within 1 year [7]. The number of international tourists visiting Japan has increased about six-fold since the start of the twenty-first century, and the countries of origin for some tourists include those with high TB prevalence rates [5,8]. Similar to Western countries, TB cases among foreign-born individuals have tended to increase in Japan [5].

## Case presentation

**Figure 1:**
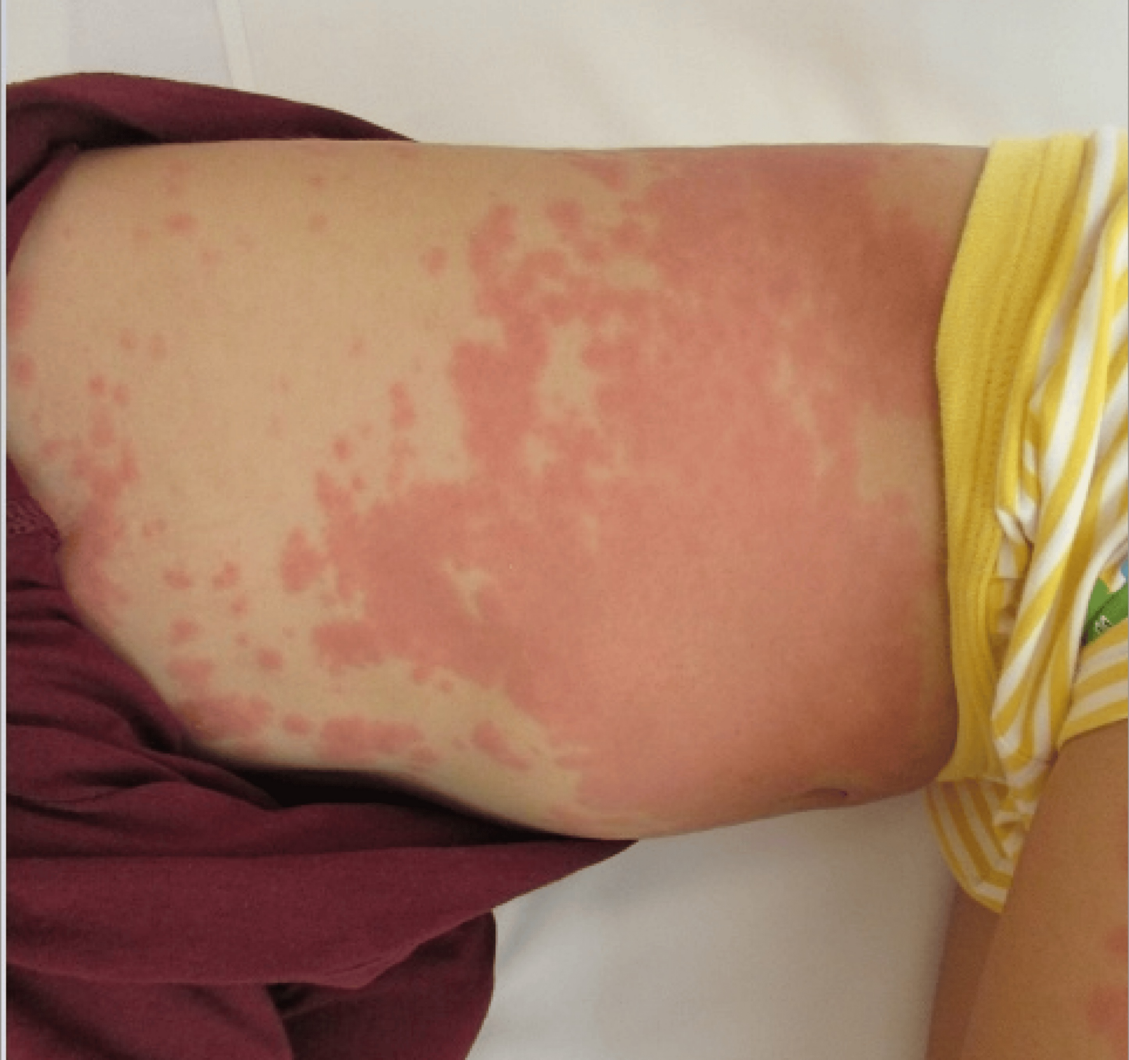
Infiltrative erythema on the body trunk, a typical symptom of Kawasaki disease.

A 56-month-old Chinese boy weighing 18.0 kg was brought to our hospital suffering from a fever lasting six days, maculopapular rash, swollen glands in the neck, red cracked lips, strawberry tongue, red inside the mouth, swollen and red limbs, and red bloodshot eyes (Figure 1).

**Figure 2:**
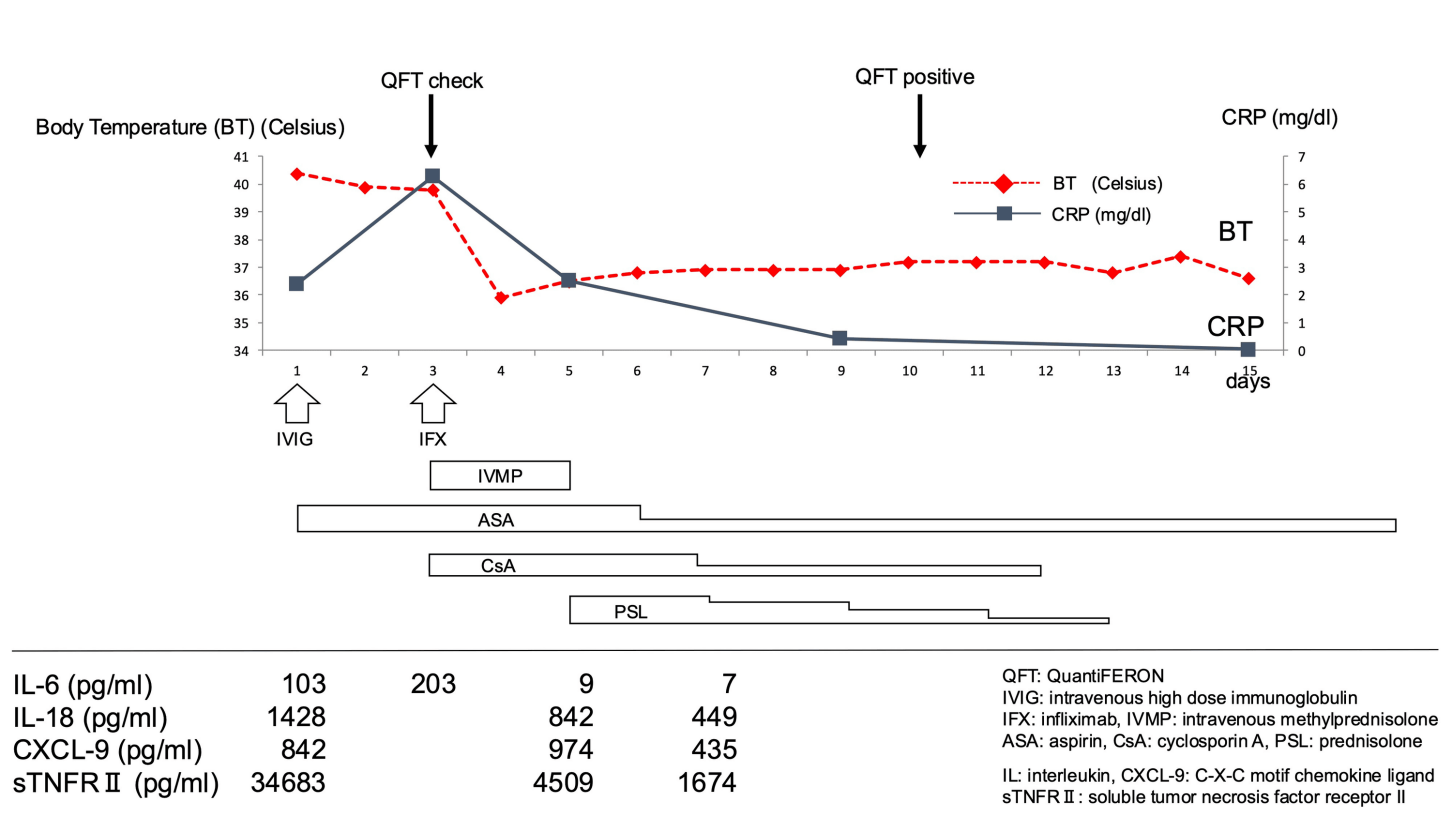
Clinical course of the acute phase of Kawasaki disease, including inflammatory markers and treatments.

He had been previously vaccinated, including with the Bacillus Calmette-Guerin (BCG) vaccine, according to China’s plan. His blood pressure was 82/31 mmHg, his pulse was 140 beats/minute, his respiratory rate was 30 breaths/minute, his tympanic temperature was 40.4°C, and his oxygen saturation was 100% on room air. Laboratory findings showed a WBC count of 5700 /mL, an absolute neutrophil count of 4161/mL, an absolute lymphocyte count of 570/mL, a hemoglobin level of 12.7 g/dL, a platelet count of 93,000/mL, an elevated C-reactive protein (CRP) level of 2.39 mg/dL, a decreased sodium level of 127 mmol/L, a mildly elevated aspartate aminotransferase level of 57 U/L, a decreased albumin level of 3.3 g/dL, and a very high inflammatory cytokine level (Figure 2). N-terminal pro-brain natriuretic peptide (NT-proBNP) and D-dimer levels were elevated (1277 pg/mL and 4.29 mg/mL, respectively). The patient's electrocardiogram exam showed good cardiac contraction and perivascular echo brightness of coronary arteries, without coronary artery dilation or pericardial effusion. Vital measurements and intravenous fluid replacement therapy were performed as initial treatment. The patient was diagnosed with KD on the 6th day of fever and administered an intravenous injection of immunoglobulin (IVIG: 2 g/kg) and aspirin (50 mg/kg). However, the high fever, conjunctival injection, painful left neck swelling, and rash persisted after IVIG administration. Follow-up laboratory results and cytokine analysis revealed the following: WBC count, 4500/µL (polymorphonuclear leukocytes, 73.9%; lymphocytes, 17.3%); hemoglobin, 11.0 g/L; platelets, 110,000/mL, CRP, 6.28 mg/dL, sodium, 130 mmol/L; and NT-proBNP, 18309 pg/mL (Figure 2).

**Figure 3:**
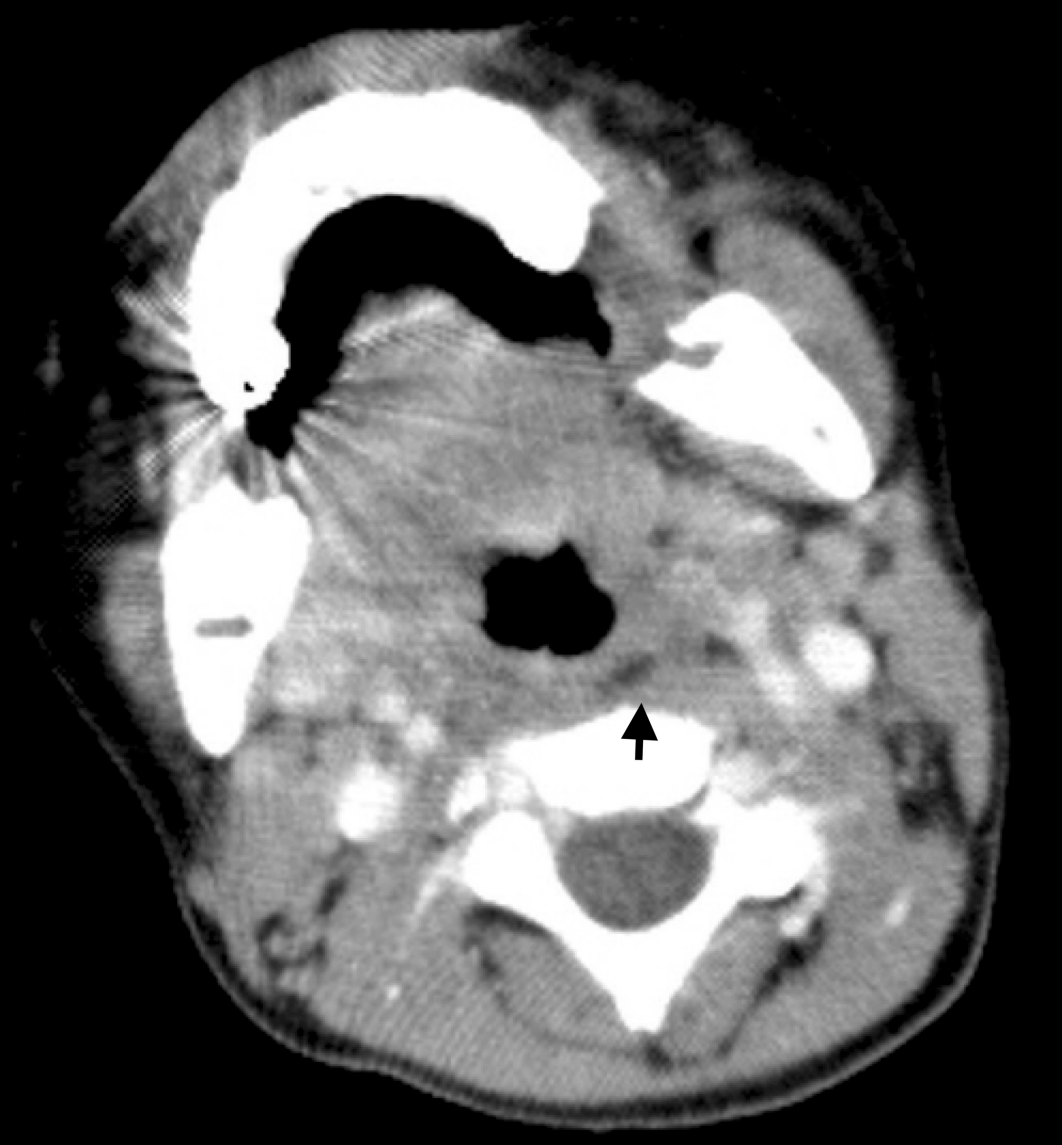
Enhanced computed tomography of the patient's neck revealed retropharyngeal edema. The black arrow indicates edema in the posterior wall of the pharynx.

On the 3rd day of hospitalization, anti-TNFα treatment consisting of IFX (5 mg/kg), intravenous methylprednisolone pulse (IVMP: 30 mg/kg), and CsA (5 mg/kg) (IFX+IVMP+CsA) was administered. On the same day, enhanced computed tomography (CT) revealed posterior retropharyngeal edema and bilateral cervical lymphadenopathy without evidence of tuberculosis (Figures 3).

Suspecting a *Mycobacterium tuberculosis* infection, we submitted a *Mycobacterium tuberculosis*-specific protein test (QuantiFERON-TB Gold: QFT). Although no bacteria were isolated from a throat swab or blood cultures performed at the first visit to our hospital, administration of infliximab (IFX), intravenous methylprednisolone pulse (IVMP), and CsA led to a rapid clinical improvement and resolution of fever and other signs and symptoms, as well as reduced cytokine levels. The amount of aspirin (ASA) was reduced on the 6th day of hospitalization and CsA and prednisolone were tapered and discontinued on the 12th and 13th days of hospitalization, respectively (Figure 2). The patient displayed desquamation of the skin of the second right finger and was discharged on the 14th day of administration with no findings of myocardial damage or coronary artery dilatation, based on the results of echocardiography and a negative CRP level of 0.05 mg/L. At follow-up 78 weeks later, echocardiography of the coronary arteries was normal.

The QFT test turned out to be positive on the 10th day of hospitalization. The patient was diagnosed with LTBI based on the absence of respiratory symptoms and suspicious findings on CT scans for pulmonary TB, as well as the absence of pathological agents in his sputum culture. The boy had a history of contact with a TB patient two months prior to the onset of KD symptoms, which included contact with a TB patient during mealtime in China. We initially treated the patient with IFX+IVMP+CsA for KD, but we are currently managing the condition with oral rifampicin (RFP: 15 mg/kg) instead of isoniazid, because isoniazid-resistant tuberculosis infections are prevalent in China. The patient and his guardians have been informed that it will be necessary to maintain this treatment for 24 weeks. While he was in the hospital, his Chinese mother, who did not speak Japanese well, was the only one accompanying him. During the COVID-19 epidemic, changes in escorts or visits by other family members were prohibited; thus, we were unable to collect sufficient information from the patient's mother regarding his medical history, including his contact with TB-infected individuals. His medical expenses amounted to $18,000, which was treated as an out-of-pocket expense. At follow-up 78 weeks later, the patient was still free of TB and reported no adverse events.

## Discussion

This study draws attention to an unusual clinical situation in which a boy traveling from abroad was found to have LTBI after having been diagnosed with KD and treated with complex immunosuppressive therapy, including anti-TNFα therapy. Currently, data on TB disease in children receiving anti-TNFα treatment are very limited. In the largest pediatric case series to date, which comprised a total of 19 patients who were diagnosed with TB disease during treatment with anti-TNFα drugs, all the patients presented with pre-defined severe disease, and one was diagnosed only during a post-mortem examination. Of the 18 patients who were alive at presentation, 14 had miliary TB, and four had central nervous system involvement, both of which are associated with high levels of morbidity and mortality. This predilection for severe TB manifestations in children on anti-TNFα biologics is further documented in several reports and small case series [9]. As far as we know, this is the first reported case of LTBI in a boy who received anti-TNFα antibody therapy after being diagnosed with KD. The risk of developing TB infection increases by 4.0 times, 2.8-7.7 times, and 2.0-3.0 times with IFX use, oral prednisolone use, and other immune agent use, respectively [10-12]. It is unclear how much the risk increases when risk factors overlap, as in our case. The pathogenesis of KD is not well understood, and further studies are needed to develop novel treatments for refractory KD and LTBI in immunosuppressed patients, if predisposing factors exist. Future challenges include determining to what extent the administration of multiple immunosuppressants increases the risk of developing TB, and establishing how much immunosuppressant can be safely used during the acute phase of KD when TB infection is suspected. Foreigners who get sick while traveling face a variety of problems, including medical costs, no health insurance, and poor communication [13,14]. An analysis of medical prices for foreign tourists in Japan showed that these prices are 1.22 to 3.66 times higher for inpatients with appendicitis and femoral fractures than for those in Japan covered by Japan’s public health insurance plans [14]. If foreign residents visiting a medical institution cannot speak Japanese well, they might not be able to explain clearly their symptoms or understand what the doctor is saying. In fact, a previous study reported that 50% of foreign residents had trouble communicating in hospitals, indicating that they experienced difficulties when visiting a medical institution due to “language barriers” [13]. To avoid excessive financial burden when traveling abroad, tourists should be advised to get medical insurance before they travel [15]. Active use of translation applications and the active introduction of medical interpreters should be considered as countermeasures for overcoming the language barrier in medical institutions [13,16].

## Conclusions

Cases of TB diagnosed in pediatric patients treated with anti-TNFα drugs have been scarcely reported. In the case presented, a patient who was treated with immunosuppressive therapy, including anti-TNFα treatment after being diagnosed with KD, was later diagnosed with LTBI and underwent TB treatment.
